# Turning the EU’s agricultural vision into environmental action: A performance-oriented CAP after 2027

**DOI:** 10.1007/s13280-025-02281-y

**Published:** 2025-10-29

**Authors:** Katharine Heyl, Beatrice Garske, Jessica Stubenrauch, Knut Ehlers

**Affiliations:** 1 Department for Environmental and Planning Law, Helmholtz-Centre for Environmental Research, Permoserstraße 15, 03418 Leipzig, Germany; 2 Section Agriculture, German Environment Agency, Wörlitzer Platz 1, 06844 Dessau-Roßlau, Germany

**Keywords:** CAP, Common Agricultural Policy, EU Vision for Agriculture and Food, Multiannual Financial Framework, Pay for performance, Policy recommendations

## Abstract

The agricultural sector of the EU is affected by several political and economic crises and dissatisfaction with policy has been voiced loudly. Against this background, the EU Commission presented a *Vision for Agriculture and Food* which emphasizes competitiveness, food security, and simplification. This News article critically analyses the *Vision*’s references to the future Common Agricultural Policy (CAP) in regards to environmental challenges and proposes alternative policy recommendations. The analysis shows that the *Vision* prioritizes income support and reduced bureaucracy while neglecting environmental protection. However, to ensure long-term food security and environmental resilience, (1) a ‘pay for performance’ approach on Member State level, (2) the expansion of results-oriented measures and (3) the promotion of circular economy principles on farm level are needed.

## Introduction

The EU Commission presented a *Vision for Agriculture and Food* in February 2025 (European Commission 2025e). The Vision is embedded in a policy context that is shaped by a reform of the Common Agricultural Policy (CAP), Union wide farmers’ protests and discussions on the Multiannual Financial Framework. It therefore does not come as a surprise that the Vision’s focus—in sharp contrast to its predecessor’s focus on sustainability, i.e., the Farm to Fork Strategy,—places a heavy focus on competitiveness, (food) security, and simplification. Overall, the Vision vaguely aims at a thriving and diverse agricultural sector.

The Vision establishes several references to the CAP which is the central political element in shaping agriculture at EU level. Given the dramatic state of agricultural landscapes in the EU, the subsidies of the CAP have been widely criticized for their lacking or even counter-beneficial impact on climate change and biodiversity loss (Pe’er et al. 2022; Guyomard et al. 2023). In fact, the (global) food system and intensive agricultural practices are the primary drivers of biodiversity loss and contribute significantly to climate change and soil degradation (Vieira et al. 2024).

Evident is that policy coherence between CAP subsidies and EU environmental objectives as established by the Water Framework Directive, the Nitrates Directive, and the Nature Restoration Regulation is insufficient just like their contribution to global environmental objectives of the Convention on Biological Diversity and the Paris Agreement (European Court of Auditors 2024). To counteract this mismatch, an effective agricultural policy which transforms agricultural systems toward sustainability is needed. In line with the Brundtland Commission’s definition of sustainability, sustainable agriculture is understood as a way of farming that meets the needs of the present generation without compromising the ability of future generations to meet their needs (World Commission on Environment and Development 1987).

Against this background, the aim of this News article is to provide policy recommendations for the future CAP to ensure environmental protection and compliance with environmental policy objectives. These recommendations build on a critical analysis of the EU Vision for Agriculture and Food.

## Status quo of the Common Agricultural Policy

The environmental impact of EU agriculture is significantly determined by the design of the CAP because the CAP continues to be the central policy instrument for the EU agricultural sector since its adoption in 1962. The CAP is currently composed of two pillars which are funded by two separate funds.

To receive direct payments of the first pillar (European Agricultural Guarantee Fund), farmers have to comply with Conditionality. Conditionality subsumes nine standards for good agricultural and environmental conditions (GAECs) and several statutory management requirements, i.e., obligations of related EU policies such as the Nitrates Directive. Besides that, famers can voluntarily participate in eco-schemes under the first pillar which account for 25% of the funding.

The second pillar of the CAP (European Agricultural Fund for Rural Development) seeks to support rural development and focusses on strengthening the competitiveness of the agricultural sector and the economic situation in these areas. In contrast to the first pillar, measures of the second pillar have to be co-funded by Member States and at least 35% of the funding has to be allocated for voluntary ‘Climate and Environmental Measures’ (e.g., support for organic farming).

The national implementation of the CAP is established in Strategic Plans that Member States have submitted to the Commission at the beginning of the funding period. Through this approach, the EU Commission aimed to give Member States the flexibility to adapt the CAP’s framework to ‘national circumstances and enhance performance’. Indicators to assess the achievement of the CAP objectives are related to outputs, results, impacts, and context (European Commission 2021 Annex I). However, despite the introduction of environmental standards and support measures, the environmental performance of the CAP remains inadequate (European Court of Auditors 2024). What’s more, farmers’ protests have led to an easing of Conditionality in 2024. Farmers are e.g., no longer obliged to dedicate a minimum part of arable land to non-productive areas to receive direct payments (European Commission 2024b). Hence, previously at length negotiated environmental standards under the first pillar were rolled back (see e.g., Hristov et al. 2020; Heyl et al. 2021).

## EU Vision for Agriculture and Food and the future CAP

This section summarizes the Vision’s links to the future CAP and to this end adopts the structure of the Vision.

### The CAP and income

The Vision clarifies that the CAP ‘remains essential to support farmers’ income’. To more fairly and better target CAP subsidies, the Commission proposes to (1) aim support at farmers actively engaged in food production, farm economic vitality, environmental preservation, those ‘who need it most’, i.e., ‘farmers in areas with natural constraints, young and new farmers, and mixed farms’, (2) consider extending simplified income support measures for small farms and (3) consider degressivity and capping.

The Commission furthermore aims to streamline and simplify payments for ecosystem services, adopt a ‘strategic approach’ for CAP implementation and rely on ‘basic policy objectives’ and ‘targeted policy requirements’. Moreover, the Vision emphasizes that the CAP will continue to provide investment support to enhance the competitiveness, sustainability and resilience of the farming sector. Member States should be given more responsibility and farmers more flexibility. Conditionality will be simplified. Overall, the CAP ought to move away from conditions to incentives.

### The CAP for a competitive and resilient sector

The Vision announces measures to better equip farmers to manage political and environmental risks including climate change. The future CAP will provide targeted measures and investments to enhance the resilience of the sector: ‘More ambitious transformational changes’ are needed for unsustainable production processes. To this end, the Vision vaguely introduces new local strategies and refers to research and innovation. Overall, to adjust to climate change, agricultural policies will have to be tailored and support climate-resilient practices.

The Commission furthermore aims to limit the regulatory burden of farmers which is expected to increase competitiveness. In line with the Commission’s overarching simplification efforts, agricultural policy will henceforth be simplified. Without naming the CAP, the Vision points out that ‘one-size-fits-all approaches’ and detailed on farm practices make a bad fit with a highly diversified sector. A simplification package is announced (see for details on the published policy: European Commission 2025c).

### The CAP and nature

The Vision points out that food production and nature are closely linked and that combatting climate change and environmental degradation will benefit the sector but also requires its contribution. It states that ‘the ecological transition must carefully integrate economic and implementation challenges, as well as the need for a just transition in social terms’. The Vision concludes (again) that well-tailored and targeted approaches, including nature-based solutions, are needed.

While expecting that the sector will achieve its emission cuts to the 2030 climate target, the Vision remains vague on policies. It states that, alongside a livestock workstream and technological advances, the future CAP will ‘assess’ how to support farmers in reducing greenhouse gases. To farm nature-friendly, ‘implementation, streamlining, and enforcement of existing legislation and using incentives’ must be improved. Farmers require more effective tools such as better targeted CAP support, tailored advice and a more ‘agile regulatory environment’. Lastly, as farming relies on healthy soils, the Commission will support practices that contribute to soil health including support for organic farming and advisory services.

## A critical analysis of the vision’s links to the future CAP

A legislative proposal for the future CAP was presented in July 2025 (European Commission 2025i). However, the new CAP’s design and how ‘a clearer balance between regulatory and incentives-based polices’ will exactly look like remains open since the trilogue—the negotiation process between European Commission, European Parliament and Council of the European Union—is yet to start. Still, this section discusses the Vision’s reference to the future CAP and thereby includes recent policy developments.

First of all, the Vision suggests that the future CAP will continue to focus on income support which aligns with von der Leyen’s political guidelines (von der Leyen 2024) and was underscored in the recently published proposal for a new CAP that exclusively ringfences the income support budget (European Commission 2025i).

The Vision does not announce new environmental measures and remains vague on existing instruments which stands in contrast to the negative environmental developments in agricultural landscapes and environmental policy objectives. The Vision furthermore fails to discuss biodiversity loss and establishes no reference to the Nature Restoration Regulation. In doing so, it excludes an essential environmental issue.

Failing to incorporate strong environmental measures and biodiversity loss is supplemented by the Commission’s plan to ‘simplify’ Conditionality. Hence, the certainly questionable current environmental impact of CAP income support (Kortleve et al. 2024) will likely be further weakened.

Simplifying Conditionality sits in a broader political context where ‘simplification’ and ‘streamlining’ policy measures are en vogue (see e.g., European Commission 2025d). While reducing administrative burdens is important, ‘simplification,’ and ‘streamlining’ likely also serve as fig leaves to (further) reduce environmental protection measures and subordinate environmental protection to competition and (food) security. As shown above, recent efforts to simplify conditionality actually led to the abolition of environmental standards rather than simplification for comparable environmental effects. The same applies in large parts to the recently published CAP simplification package which proposes to e.g., erode protection standards for permanent grasslands (European Commission 2025h).

Simplification is furthermore likely to be accompanied by a partial renationalization of the CAP budget and environmental obligations (European Commission 2025g). For example, the proposal for the new Multiannual Financial Framework does not include a fixed budget for environmental measures as opposed to the current CAP (European Commission 2025f Annex). As a consequence, Member States could prioritize CAP environmental measures differently, i.e., allocate more or less of their budget to them. This could counteract a level playing field for EU farms and fuel a race to the bottom. Moreover, the proposed ‘protective practices’ attached to the income support (‘farm stewardship’) (European Commission 2025a) are vaguer and less prescriptive than the current obligations of Conditionality which would again weaken the environmental impact of income support.

That being said, the Vision’s repeated emphasis to better target CAP subsidies to local needs could be an important feature to ensure high environmental performance of subsidies (Zindler et al. 2024). Likewise, the Vision’s reference to the CAP as an instrument to make farming more resilient to changing climatic conditions and reduce emissions is also an important, although vague, aspect. Besides that, focusing income support on mixed farms and ‘farmers in areas with natural constraints’ could actually have some indirect beneficial environmental effects. Mixed farms are potentially better suited to implement a (nutrient) circular economy compared to pure livestock or arable farms (Leite et al. 2024), and farming on marginal land can help maintain agricultural activity and prevent land abandonment (Reger et al. 2009).

The continuation of investment support appears useful if these measures clearly contribute to achieving environmental policy objectives as established by the Water Framework Directive, the Nitrates Directive, and the Nature Restoration Regulation. Also, reducing bureaucracy may free up resources to better design targeted subsidies and dialogue may improve the design, uptake, and acceptance of environmental measures (Reichenspurner et al. 2024).

Lastly, the Vision correctly recognizes that critical dependencies of the EU agricultural sector must be reduced to be more resilient in the face of shocks. Pursuing increased self-sufficiency has the potential to minimize market fluctuation risks (Sánchez et al. 2022). At present, however, the EU agricultural sector is highly reliant on external inputs such as protein feed and food or fertilizers (European Commission 2024a).

## Policy recommendations for the future CAP

### (Food) security, competitiveness, and simplification must not be pursued at the expense of environment and climate protection

Overall, a prerequisite for successfully realizing the goals of the Vision and EU environmental policy objectives is a high level of environmental and climate protection. Preserving healthy soils, sufficient and clean water, and a stable climate are preconditions for any farming activity and yield stability. Thus, (food) security and (crisis) resilience can only be achieved through farming within planetary boundaries. Below, we firstly propose policy amendments at the Member State level and secondly at the farm level.

### Fuel a race to the top, not to the bottom

Linking CAP support to environmental performance is crucial. This needs to be further strengthened on farm level (see sections below) but could just as well be implemented on Member State level. While an increase of national responsibility on how CAP money is spent seems likely (see above), steps need to be taken to avoid a race to the bottom regarding the environmental CAP standards between Member States. We propose to develop a mechanism that couples national CAP budgets to Member States’ environmental ambition.

This mechanism could be integrated into the National and Regional Partnership Plans which the Commission proposed alongside the future CAP and Multiannual Financial Framework. These Plans subsume several policy areas including cohesion, fisheries, climate change, defense, and agriculture and the Commission plans to integrate the CAP into these overarching Plans (European Commission 2025j).

**Fig. 1:**
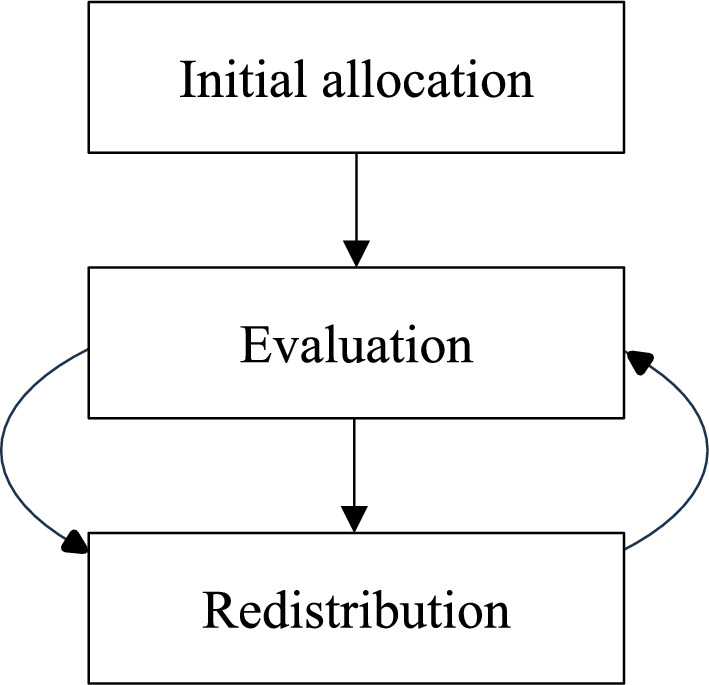
‘Pay for performance’ process

The Commission proposes to allocate the funds for these Plans to Member States according to a formula (European Commission 2025b Annex I). This formula covers the funding for the entire Plans and hence multiple policy areas. And while it incorporates e.g., GPD and population, it does not include a performance component (see for details Matthews 2025). Against this background, we propose a separate allocation mechanism for CAP funds based on environmental performance. Such a ‘pay for performance’ mechanism could roughly look like this (Fig. 1):

Step one (initial allocation): Member States start the new CAP funding period with the budget dedicated to agriculture within their National and Regional Partnership Plan.

Step two (evaluation): The environmental performance of Member States’ agricultural budget is evaluated.

Step three (redistribution of funds): Member States that excel are granted additional funds, while Member States that allocate agricultural payments with comparatively little added value for the environment will get less money.

Step two and three are repeated in regular intervals such as every 1–2 years.

In order to perform a transparent evaluation, a benchmarking at EU level has to be designed. To this end, the performance framework of the current CAP could serve as a basis (European Commission 2021 Annex I). There, impact indicators include, e.g., reducing nutrient leakage and enhancing carbon sequestration. The corresponding results-based indicators are “land share that is subject to improved nutrient management” and “increasing carbon storage in soil and biomass”. Still, more research is needed to comprehensively evaluate Member States’ budget performance.

In fact, the framework for a ‘pay for performance’ mechanism overall requires further research to also discuss potential challenges such as decreased budget planning security, transaction costs, political feasibility as well as bureaucratic workload and potentially disturbed level playing fields. Nevertheless, such a ‘pay for performance’ mechanism could fuel innovation and ambition to develop new and attractive environmental measures and give less successful Member States inspiration and incentives to learn from more successful countries.

### Improve the competitiveness for sustainable farming

Achieving a high performance at the Member State level requires effective measures at the farm level. Thus far, farms with a good environmental performance are placed at a competitive disadvantage unless being compensated for the production of common and public goods. Although sustainable farming is economically viable in the long term because, e.g., more crop diversity and healthy soils increase resilience to extreme weather events, sustainable farming often requires investments, e.g., in diversification (Sánchez et al. 2022). Currently, non-sustainable farms are thus privileged despite causing higher environmental costs. Therefore, focusing CAP support on verifiable farm environmental performance remains indispensable to assure a level playing field for sustainable farming.

### Focus public support on environmental performance rather than categories

The Vision proposes to allocate CAP support to farms based on certain categories such as small farms or young farmers. The overall aim is to ensure support is ‘directed toward those farmers who need it most’ (European Commission 2025e). Yet, scientific evidence that these farms perform better environmentally is lacking. Furthermore, focusing public support on these farms does not necessarily unlock transformative potential towards greater sustainability. Providing subsidies based on farm-specific environmental performance therefore likely achieves a higher environmental impact compared to a farm category-oriented approach (Wätzold et al. 2016). Thus, focusing public support toward those farmers who deserve it most will be more beneficial for society in the long run.

### Enable entrepreneurial freedom wherever possible

The future CAP intends to focus on incentives rather than regulations. Although no farmer is legally forced to accept CAP subsidies and comply with its requirements, CAP funding is of vital importance for many farms. Simultaneously, farmers cooperation is essential for the environmental performance of the CAP. Hence, offering an attractive and easy to implement environmental toolbox to farmers is necessary. However, the current voluntary toolbox of the CAP (e.g., eco-schemes) is frequently not suited to meet farmers demands and uptake by farmers remains low in some Member States (European Court of Auditors 2024). To address this issue, shifting from action-oriented measures to result-oriented measures is one promising option (Burton and Schwarz 2013; Bartkowski et al. 2021). For instance, while action-oriented measures on nutrient losses are requirements on how to apply which and how much fertilizer at what time, a result-oriented measure could be a nutrient balance on farm level. Farmers would then be incentivized by rewarding reduced recorded nutrient losses into the environment, but the choice of the concrete measures remains within their entrepreneurial decision scope. This could make environmentally friendly farming more attractive and the CAP thus more effective.

### Enhance resilience by supporting circular economy principles

In addition to climate-adapted, environmentally friendly farming, agricultural practices based on circular economy principles contribute to increased resilience. In general, mixed crop-livestock systems are potentially less dependent on external inputs, although local environmental conditions have to be considered (Leite et al. 2024). For example, if feed is grown on the farm or in the region, less protein feed needs to be imported. This reduces critical trade dependencies while having advantages for local soils, particularly with regard to crop diversification, nitrogen fixation, and soil loosening due to legume cultivation (European Commission. Joint Research Centre 2024). Furthermore, the use of recycled fertilizers instead of mineral and synthetic fertilizers decreases dependency from imported phosphorus fertilizers and energy-intensive nitrogen fertilizers (European Commission 2022). Thus, the future CAP must target support toward environmental and climate protection as well as circular economy principles.

## Conclusions

The policy recommendations for a future CAP with enhanced environmental performance are derived from the EU *Vision on Agriculture and Food*. As such, these recommendations build on and extent an environmentally weak policy foundation. They thus reflect a very challenging political context for environmental protection policies. Yet, there is broad consensus among scientists that a truly transformative reform would be needed to ensure policy coherence between the CAP and EU and international environmental policy objectives. To this end, EU funds have to be allocated to achieve good environmental performance. However, the recently published proposals for the future CAP paints a rather different picture.
